# Light photon flux density affects ethanol-mediated drought avoidance in cassava (*Manihot esculenta* Crantz)

**DOI:** 10.5511/plantbiotechnology.25.0426a

**Published:** 2025-12-25

**Authors:** Anh Thu Vu, Yoshinori Utsumi, Chikako Utsumi, Daisuke Todaka, Quynh Do Thi Nhu, Xuan Hoi Pham, Motoaki Seki

**Affiliations:** 1 National Key Laboratory for Plant Cell Biotechnology, Agricultural Genetics Institute, Pham Van Dong Road, Bac Tu Liem District, Ha Noi, Vietnam; 2 Plant Genomic Network Research Team, RIKEN Center for Sustainable Resource Science, Yokohama, Kanagawa 230-0045, Japan; 3 Plant Epigenome Regulation Laboratory, RIKEN Cluster for Pioneering Research, Wako, Saitama 351-0198, Japan; 4 Kihara Institute for Biological Research, Yokohama City University, Yokohama, Kanagawa244-0813, Japan; 5 Graduate School of Science and Engineering, Saitama University, Saitama, Saitama 338-8570, Japan

**Keywords:** cassava, drought avoidance, ethanol, light photon flux density, stomatal closure

## Abstract

Cassava is a globally important food source. Given the increasing frequency of climate change-induced drought, enhancing the drought resilience of cassava is paramount. Chemical priming can bolster tolerance to stress factors. We previously determined that pretreatment with low concentrations of ethanol enhances abiotic stress tolerance in Arabidopsis, tomato, and cassava. Nevertheless, the efficacy of ethanol treatment in complex natural settings remains to be fully explored. In this study, we assessed the impact of ethanol treatment on cassava under varying light photon flux densities (PFDs) and drought conditions. We observed that drought tolerance was enhanced by ethanol pretreatment at high (∼400 µmol photons m^−2^ s^−1^) and medium (∼60 µmol photons m^−2^ s^−1^) light PFDs but not under low light PFD (∼4 µmol photons m^−2^ s^−1^). Ethanol pretreatment under high and medium light PFDs promoted stomatal closure and drought avoidance, thereby preserving higher water content in plant tissues. Furthermore, ethanol pretreatment under these PFDs upregulated expressions of genes associated with ABA signaling and heat shock proteins (HSPs) relative to water pretreatment. In addition, starch accumulation in leaves was observed under all light PFDs with ethanol pretreatment. We hypothesize that ethanol pretreatment at light PFDs exceeding 60 µmol photons m^−2^ s^−1^ facilitates ethanol-mediated drought avoidance in cassava by activating at least three pathways: 1) ABA signaling, 2) protein folding-related response via triggering of the HSP/chaperone network, and 3) alterations in sugar and starch metabolism. Our findings support the application of optimal light PFDs to enhance the benefits of ethanol-induced drought avoidance in cassava.

## Introduction

Light is the primary energy source for photosynthesis in plants, and its intensity and quality vary continuously under the influence of weather conditions and seasonal shifts due to solar inclination (Casal 2013). Light also induces stress in plants and modulates their stress responses. Excessive and fluctuating light intensities can lead to photoinhibition and reactive oxygen species (ROS) accumulation around photosystems II and I, respectively (Roeber et al. 2021). Furthermore, light influences the expression of heat shock proteins (HSPs), which increases significantly under elevated temperatures (Roeber et al. 2021). Drought tolerance is similarly critical for mitigating negative impacts on plant performance and productivity. Drought stress induces specific signaling pathways that drive physiological and developmental changes to optimize plant water use (Fahad et al. 2017; Nakashima et al. 2014). In response to drought, plants minimize water loss by closing stomata, a process regulated by abscisic acid (ABA), whose biosynthesis is enhanced under drought conditions. Stomatal behavior is also influenced by diurnal temperature (Tallman 2004) and light cycles (Matthews et al. 2020).

Cassava (*Manihot esculenta* Crantz), a versatile crop used for food, fuel, animal feed, and industrial purposes, is notably drought tolerant (Malik et al. 2020; Utsumi et al. 2012), but prolonged drought can lead to leaf shedding and reduced growth and yield (El-Sharkawy 2004). Drought management strategies should leverage molecular tools to enhance growth and yield under limited rainfall (Muiruri et al. 2021). Chemical priming, particularly with ethanol, has shown promise in enhancing multi-stress tolerance across various crops (Bashir et al. 2025; Sako et al. 2020). For example, ethanol application improves drought tolerance in Arabidopsis, rice, and wheat by modulating ABA signaling and acetic acid biosynthesis, in turn leading to reduced water loss through stomatal closure (Bashir et al. 2022). In soybean, foliar ethanol application during drought stress increases biomass and modulates photosynthetic traits, antioxidant defense, and osmoprotectant levels, thereby enhancing drought acclimatization (Rahman et al. 2022). Ethanol treatment also mitigates ROS production induced by high light stress to enhance high light stress tolerance in Arabidopsis (Sako et al. 2021). Our previous research has indicated that ethanol treatment enhances drought stress avoidance in cassava by inducing stomatal closure through three mechanisms: 1) activation of the ABA signaling pathway, 2) a protein folding-related response via the HSP/chaperone network, and 3) changes in sugar and starch metabolism (Vu et al. 2022). Furthermore, higher leaf temperatures were detected after 5 days in cassava plants treated with ethanol under sunlight compared with those treated with water, with this initial physiological signal then leading to increased expression of heat shock protein-related genes such as *HSP70*, *HSP90*, and *HSP101* (Vu et al. 2022).

On the basis of the above findings, we hypothesized that variations in light photon flux density (PFD) may influence the effectiveness of ethanol-mediated drought avoidance through modulation of the above three pathways. In this study, we therefore evaluated the impact of light PFD on ethanol-mediated drought avoidance in cassava using high (∼400 µmol photons m^−2^ s^−1^), medium (∼60 µmol photons m^−2^ s^−1^), and low (∼4 µmol photons m^−2^ s^−1^) light PFDs. According to our results, ethanol pretreatment, coupled with high and medium light PFDs, induced stomatal closure and drought avoidance by maintaining higher water content in plant tissues. In addition, the expression of genes related to ABA signaling and HSPs was upregulated compared with water pretreatment under the same light PFDs. Conversely, the phenotypic changes associated with induced stomatal closure and related gene expression were not observed under low light PFD. These findings indicate that optimal light PFD significantly enhances the efficacy of ethanol in facilitating drought avoidance in cassava.

## Materials and methods

### Cassava cultivar and plant preparation

The African cassava cultivar 60444 was obtained from the in vitro cassava germplasm collection at the International Institute of Tropical Agriculture (IITA, Nigeria). The in vitro cassava plantlets were initially acclimatized to ambient atmospheric conditions and subsequently maintained under the following controlled greenhouse conditions: 50% relative humidity, 28°C, and 16 h of supplemental lighting using a ceramic metal-halide lamp (FEC Cera Arc Ace PRO 360W, white light 4100 K) (Iwasaki Electric Co., Tokyo, Japan). Stem cuttings, approximately 3 cm in length, were harvested from individual plants and propagated. When the cuttings reached a stem height of approximately 20 cm above the soil surface, they were transplanted into plastic pots (7.9 cm diameter × 6 cm height) filled with a soil mix consisting of vermiculite, black soil, and coconut fiber in a 2 : 1 : 2 ratio. After transplantation, the plants were cultivated under the same greenhouse conditions for an additional 2 weeks before use in experiments. For the experiments, plants were subjected to pretreatment with either 200 ml of 1% (v/v) ethanol or water (control) per 1 plant for 5 days, followed by exposure to drought stress for 12 days under three distinct light PFDs (high: ∼400 µmol photons m^−2^ s^−1^; medium: ∼60 µmol photons m^−2^ s^−1^; and low: ∼4 µmol photons m^−2^ s^−1^) in the greenhouse (50% relative humidity, 28°C, and a 12-h/12-h light/dark cycle) (Supplementary Figure S1). Pots were covered with plastic wrap to minimize water loss from the soil surface.

### Determination of water content

The relative water content (RWC) of leaves was determined according to a previously described protocol (Nishiyama et al. 2011). The 3rd leaf from each plant was harvested and its fresh weight (FW1) was recorded. The leaves were then hydrated by floating them on deionized water in a Petri dish. Post hydration, the leaves were promptly removed from the water, gently dried of surface moisture using tissue paper, and immediately weighed again to obtain the turgid weight (TW). Subsequently, the leaves were oven-dried at 80°C for 24 h and weighed to ascertain the dry weight (DW). This procedure was replicated with six biological samples. The RWC was calculated using the formula: RWC (%)=[(FW1−DW)/(TW−DW)]×100. Additionally, to assess the water content of both leaves and stems, these tissues were separately harvested from each plant and their fresh weight (FW2) was noted. These samples were then oven-dried at 60°C for 72 h and reweighed to determine the dry weight (DW). The water content of the stems and leaves was computed using the formula: WC (%)=[(FW2−DW)/FW2]×100.

### Quantification of leaf wilting

Quantification of leaf wilting was performed essentially as described by Utsumi et al. (2019). Cassava plants were placed on a rotating table, and 360-degree images were captured to assess leaf wilting under drought conditions. For each leaf, an image with the petiole parallel to the camera was selected. The midrib line was drawn from the base to the midpoint of the midrib, and the angle between this line and the vertical axis was measured using ImageJ as an indicator of drought-induced wilting. Based on the angle, we defined leaves which have greater than 50 degree angle, leaves that have less than 50 degree angle and dried leaves that fall off stems as unwilted leaves, wilted and dried leaves, respectively.

### Thermal imaging

Thermal images were captured using an R500EX-S infrared camera equipped with a standard lens (Nippon Avionics Co., Ltd., Kanagawa, Japan). To observe leaf temperature over a broad range, the camera was adjusted at an angle of approximately 50° and set at approximately 200 cm above the leaf canopy.

### Stomatal aperture analysis

Stomatal samples were prepared from the epidermal layers of the 3rd leaves of plants pretreated with either ethanol or water under varying light PFDs (high, middle, and low). From the abaxial side of each leaf, 3 to 5 epidermal pieces were carefully peeled and immediately immersed in an observation solution comprising 5 mM MES-KOH (pH 6.0), 10 mM KCl, and 1 mM CuCl_2_. A small drop of solution was applied using tweezers. Stomata were observed and photographed using a digital microscope (VB-7010) (Keyence, Osaka, Japan). Stomatal aperture measurements were conducted and analyzed using ImageJ software.

### Measurement of sucrose, glucose, fructose and starch contents in leaves

Leaves ranging from the first to the third position from the top of the stems were collected from plants pretreated with either 1.0% ethanol or water for five days and subsequently subjected to drought stress for 6 and 12 days. Sampling was conducted between 10:00 and 11:30 AM, and the leaves were immediately frozen in liquid nitrogen. This procedure was replicated across four biological samples. The frozen leaves were pulverized in liquid nitrogen using a Multi-Beads Shocker system (YASUI KIKAI, Osaka, Japan). After the leaf samples (fresh weight: 100 mg) were boiled for 10 min in 500 µl of 80% ethanol, they were dried in a vacuum. The dried samples were resuspended in 0.3 ml of distilled water and centrifuged at 3000×g for 10 min. This process was repeated three times. The supernatants from all extractions were mixed. The concentrations of sucrose, glucose, and fructose in the supernatant were quantified using an enzymatic method (Utsumi et al. 2011).

### Gas exchange measurement

The rates of photosynthesis (µmol CO_2_ m^−2^ s^−1^), stomatal conductance to H_2_O (mmol H_2_O m^−2^ s^−1^), and transpiration rate (mmol H_2_O m^−2^ s^−1^) were assessed using the second and third fully expanded leaves from each of six biological replicates. Measurements were conducted between 10:00 and 12:00, quantifying the values per 1 cm^2^ of leaf surface area. A portable infrared gas analyzer, LI-6400 (LI-COR Ltd., Nebraska, USA), equipped with a conifer chamber, RGB light 6400-22L, was utilized. The chamber conditions were maintained at a CO_2_ concentration of 400 ppm and a temperature of 28.0°C.

### Total RNA extraction

Leaves from first to third positions from the top of the stems were collected from plants pretreated with either 1.0% ethanol or water for 5 days. Leaves were sampled between 10:00 and 11:00 AM and then immediately frozen in liquid nitrogen for total RNA extraction. The frozen leaves were pulverized using a Multi-Beads Shocker system. Total RNA was extracted from 100 mg of pulverized leaf tissue as described previously (Vu et al. 2022) and then purified using Plant RNA reagent (ThermoFisher, MA, USA). To remove any genomic DNA contamination, the RNA samples were treated with DNase I (Takara, Shiga, Japan) and an RNase inhibitor for 30 min at 37°C. Subsequent purification was performed using a RNeasy Plant Mini kit (Qiagen, Hilden, Germany). The quality of the RNA was assessed via agarose gel electrophoresis. The purified total RNA samples were stored at −80°C until further analysis.

### Real-time reverse transcription-polymerase chain reaction (qRT-PCR) analysis

cDNA was synthesized from 2 µg of total RNA using 100 units of M-MLV reverse transcriptase (Nippongene, Tokyo, Japan), 1 µl of 10 mM dNTPs, 1 µl of 10 µM oligo (dT)20 primer (Toyobo, Osaka, Japan), and 1 µl of 2.5 µM random 9-mer primers in a total volume of 10 µl. The synthesized first-strand cDNA was stored at −30°C until further use. Specific primers were designed using the Primer3 program to achieve a melting temperature (Tm) of 60°C (Supplementary Table S1). Real-time RT-PCR amplifications were conducted in 10-µl reaction volumes containing 5 µl of 2x PowerUp SYBR Green master mix (ThermoFisher), 20 ng cDNA, and 1 µM each of forward and reverse primers using the StepOne Plus Real-Time qPCR system. The amplification protocol consisted of initial denaturation at 95°C for 1 min, followed by 40 cycles of 95°C for 15 s and 60°C for 60 s. *26S proteasome regulatory subunit N10* (Manes.02G137500) gene was used as an internal control (Hu et al. 2016). The experiments were replicated with four biological samples. Relative gene expression levels were calculated using the comparative Ct method. The relative expression of each gene in a sample (ΔCt) was calculated as follows: Ct (target gene)−Ct (control gene).

### Statistical analysis

The results of gas exchange, stomatal aperture, qRT-PCR, and RWC measurements of leaves were analyzed by one-way ANOVA, and differences among means were analyzed with Tukey’s method using R software.

## Results and discussion

### Ethanol-induced drought tolerance requires high or medium light PFD

**Figure 1:**
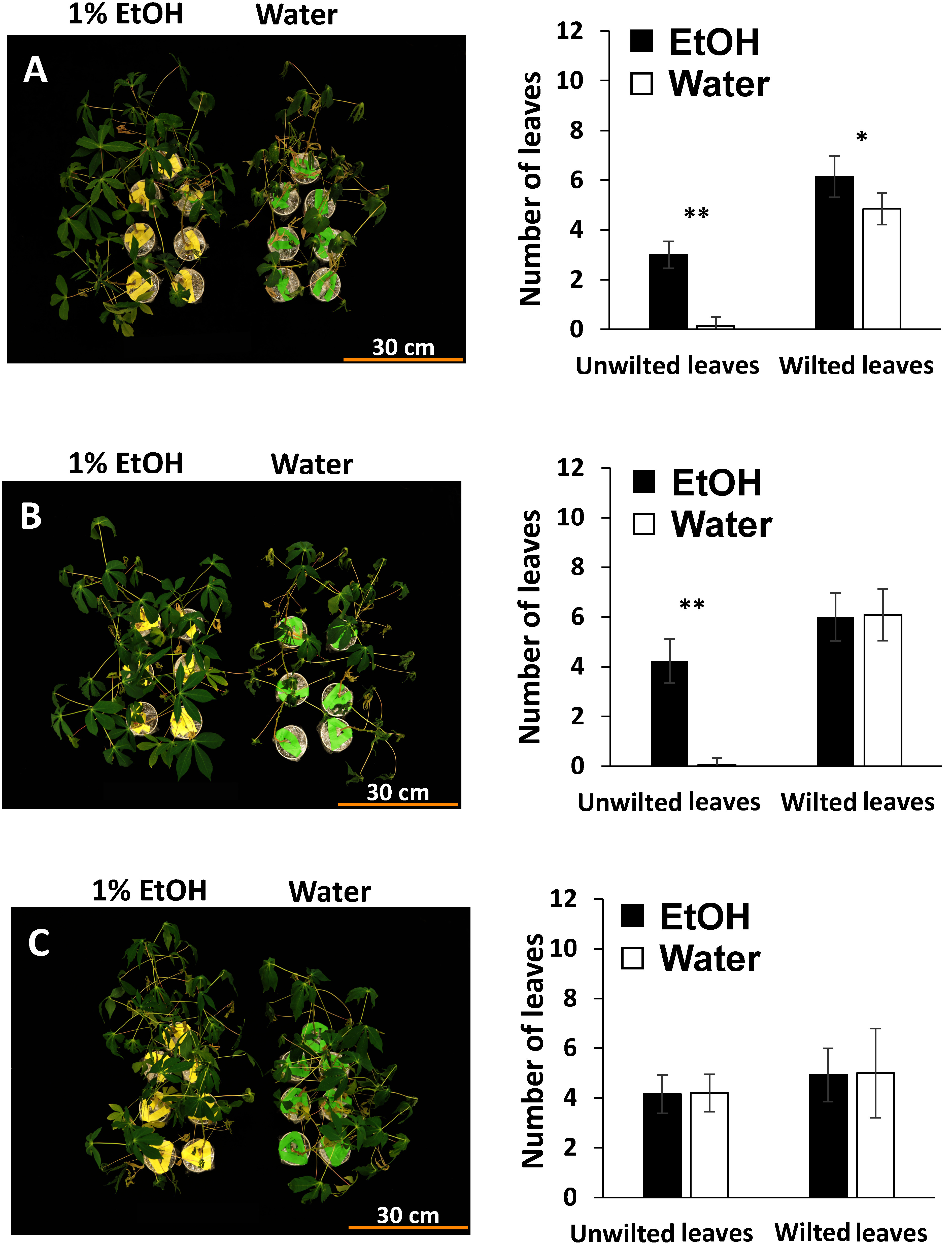
Figure 1. Representative phenotypes of cassava plants and leaf status after pretreatment with 1% ethanol or water followed by drying for 12 days under different light PFDs. Scale bar=30 cm, *n*=12. Error bars represent the mean±standard deviation (SD). Significant differences (**, *p*≤0.01 and *, *p*≤0.05) were calculated using Student’s *t*-test. Panels A, B, and C represent plants under high, medium, and low light PFDs, respectively.

In the absence of adequate moisture during drought treatment, cassava leaves tend to drop from the lower part of the stems (Utsumi et al. 2019; Vu et al. 2022). To examine the physiological effects of ethanol on cassava plants under high, medium, and low light PFDs, we evaluated phenotypes, relative water content, and soil moisture content under drought stress. Twelve days after the initiation of drought, cassava leaves pretreated with water exhibited wilting, whereas those pretreated with ethanol under high and medium light PFDs had more unwilted leaves, namely, 3–4 leaves per plant compared with the water-pretreated counterparts (Figure 1A, B). In six days after the initiation of drought, those under high and medium light PFDs had more unwilted leaves compared to those under the water treatment (Supplementary Figure 2A, B). Under low light PFD, no significant differences in the number of unwilted leaves were observed between ethanol- and water-pretreated plants under 6 and 12 days after the initiation of drought. (Figure 1C and Supplementary Figure 2C).

**Figure 2:**
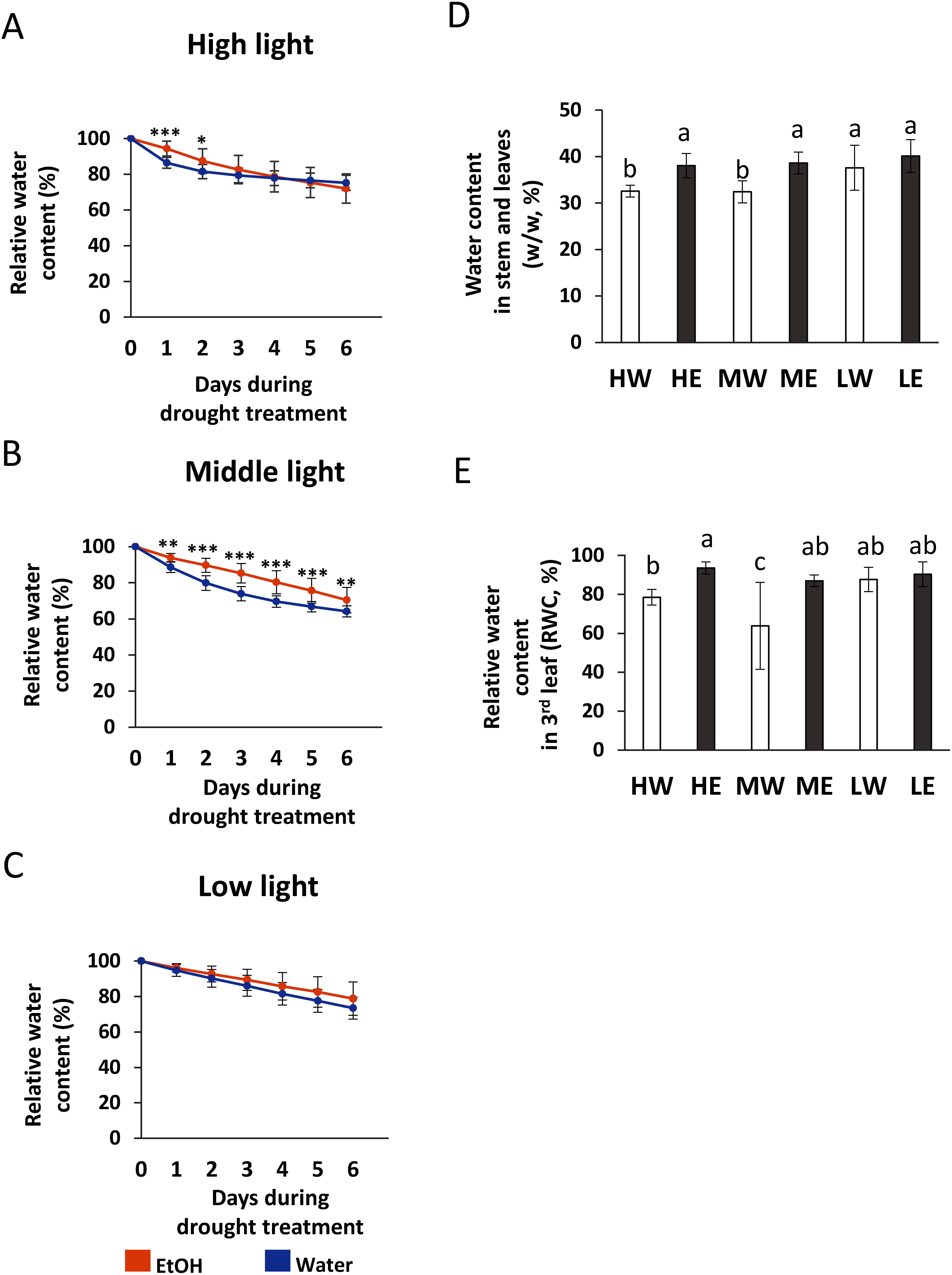
Figure 2. Decrease in water content in soil pots and relative water content in entire plants after pretreatment with ethanol or water followed by drying under different light PFDs. Panels A, B, and C show the rate of water decrease in soil pots and plant water content after the commencement of drying under high, medium, and low light PFDs, respectively, *n*=18. Error bars represent the mean±standard deviation (SD). Significant differences (*, *p*≤0.1; **, *p*≤0.05; ***, *p*≤0.01) were calculated using Student’s *t*-test. D) Water content of leaves and stems at 12 days after the commencement of soil drying under different light PFDs, *n*=7. Error bars represent the mean±SD. Bars with the same letter indicate no significant differences (*p*≤0.1, Tukey’s test). E) Relative water content of the third leaf from the top of the plant at 12 days after the commencement of soil drying under different light PFDs, *n*=9. Error bars represent the mean±SD. Treatments are indicated as follows: HW, water under high light; HE, ethanol under high light; MW, water under medium light; ME, ethanol under medium light; LW, water under low light; and LE, ethanol under low light.

In both ethanol- and water-pretreated plants, soil moisture content decreased under all three light PFDs during 6 days of drought stress treatment. Under high and medium light PFDs, however, the decrease in soil water content was less pronounced in ethanol-treated plants than in water-treated ones. During the first 3 days of drought treatment, soil moisture content in water-pretreated plants decreased rapidly, to 79.4% under high light and 73.9% under medium light, whereas the level in ethanol-pretreated plants remained higher, at 82.6% and 85.3%, respectively (Figure 2A, B). Under low light, in contrast, the decrease in soil water content between ethanol and water treatments did not change significantly, with values remaining at 90.3% and 86.1%, respectively (Figure 2C).

Water content in stems and leaves was analyzed 12 days after the onset of drought treatment (Figure 2D). Under high and medium light PFDs, the water content of stems and leaves of ethanol-pretreated plants remained higher, 38.0% and 38.6%, respectively, compared with those of water-pretreated plants, which stayed at approximately 32.5%. Under low light PFD, water contents of stems and leaves of ethanol-pretreated and water-pretreated plants were similar, 40.1% and 37.6%, respectively. At 12 days, we also measured the relative water content and phenotype of third leaves (Figure 2E). Under high and medium light PFDs, the relative water content of third leaves of ethanol-pretreated plants were significantly different from those of water-pretreated ones, whereas no differences were observed under low light PFD (Figure 2E). These results indicate that ethanol pretreatment specifically enhances drought tolerance in cassava under high and medium light PFDs, thus suggesting the existence of a threshold level of light PFD for acquiring ethanol-mediated drought tolerance.

### Stomatal closure by ethanol occurs only under high or medium light PFD

**Figure 3:**
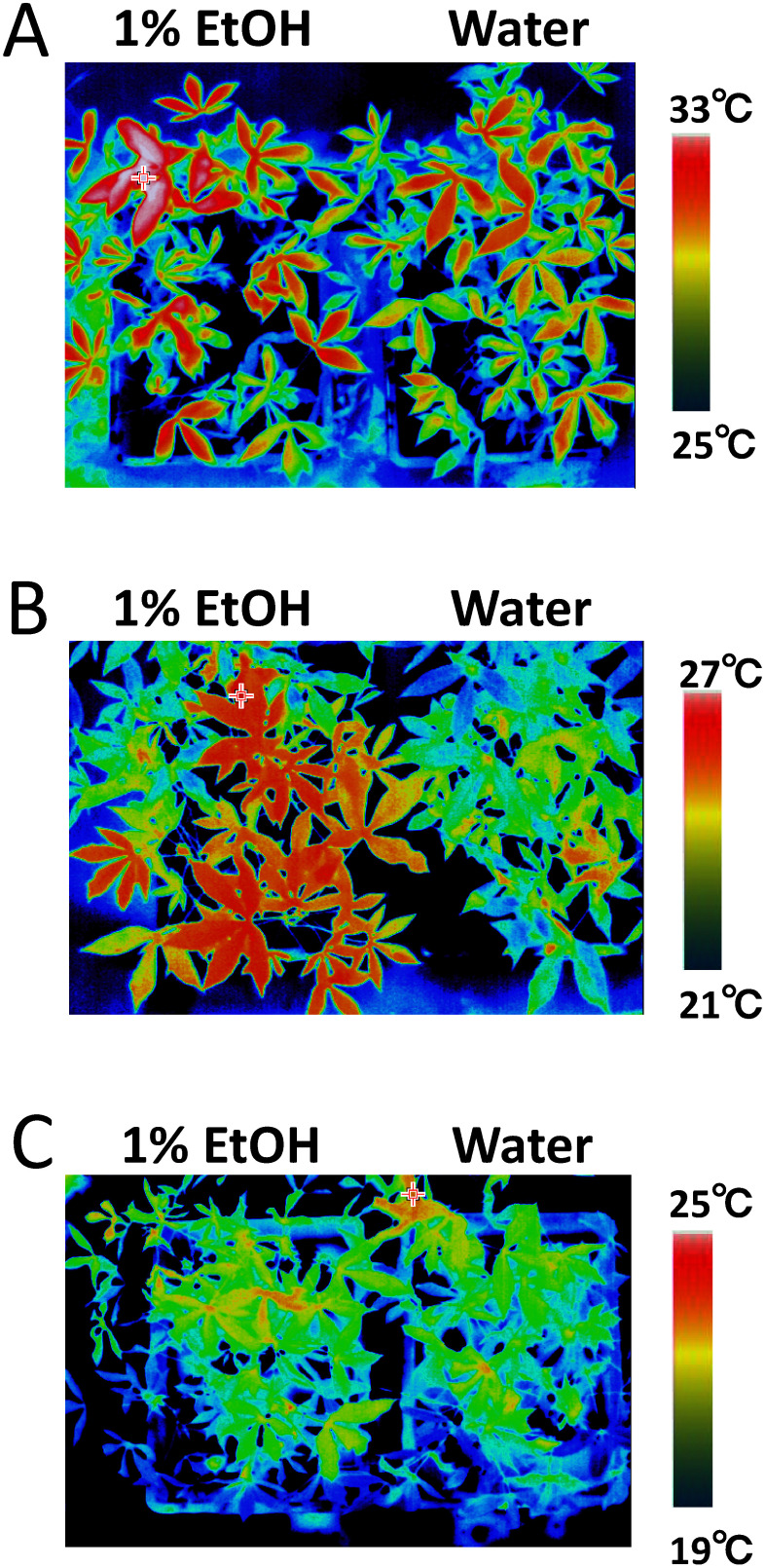
Figure 3. Thermal images of cassava plants pretreated with either ethanol or water under different light PFDs for 5 days. A) High light PFD. B) Medium light PFD. C) Low light PFD.

**Figure 4:**
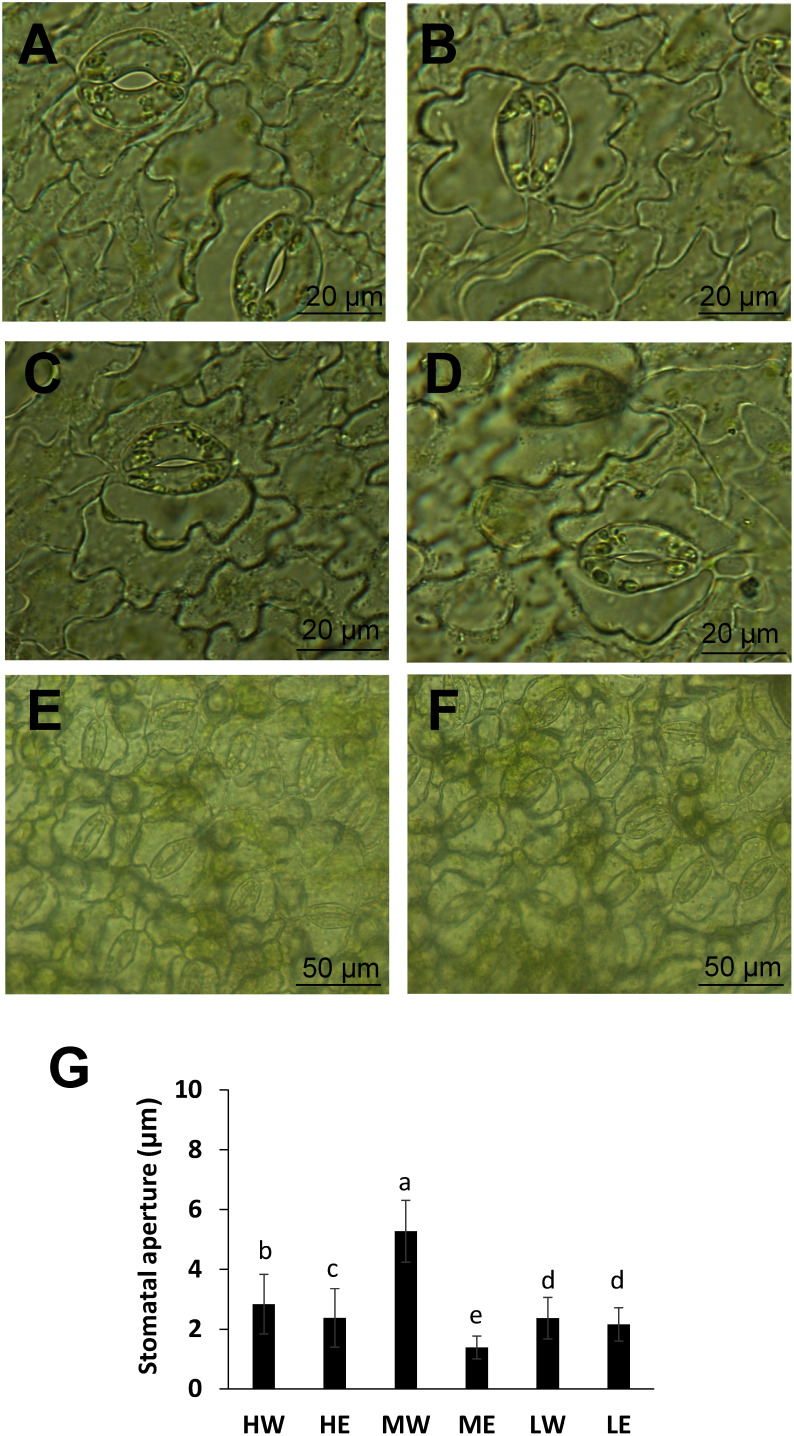
Figure 4. Effect of ethanol pretreatment under different light PFDs on stomatal aperture. A) Stomatal opening in a leaf from a water-treated plant under middle light. B) Stomatal closure in a leaf from an ethanol-treated plant under middle light. C) Stomatal opening in a leaf from a water-treated plant under high light. D) Stomatal closure in a leaf from an ethanol-treated plant under high light. Bars=20 µm. E) Stomatal opening in a leaf from a water-treated plant under low light. F) Stomatal closure in a leaf from an ethanol-treated plant under low light. Bars=50 µm. G) Stomatal aperture in plants pretreated with either ethanol or water for 5 days under different light PFDs. HW, HE, MW, ME, LW, and LE represent the treatments of water under high light, ethanol under high light, water under middle light, ethanol under middle light, water under low light, and ethanol under low light, respectively. *n*=60, Error bars represent the mean±standard deviation (SD). Bars with the same letter indicate no significant differences (*p*≤0.1), with *p*-values calculated using the Tukey test.

To investigate the effects of ethanol pretreatment on stomatal aperture under varying light PFDs, we measured leaf temperature and stomatal aperture in plants treated with either ethanol or water. Measurements were taken 5 days post-treatment under high, medium, and low light PFDs. Under high and medium light PFDs, leaf temperatures were higher in ethanol-pretreated plants compared with water-pretreated ones (Figure 3A, B). No significant changes in leaf temperature were observed under low light PFD (Figure 3C). Stomatal closure was evident in ethanol-pretreated plants; under medium light PFD, the average stomatal aperture was approximately 1.4 µm smaller than in water-pretreated plants, which averaged 5.3 µm (Figure 4A, B, G). Under high light PFD, stomatal aperture in ethanol-pretreated plants was reduced to 2.4 µm compared with 2.8 µm in water-pretreated plants (Figure 4C, D, G). Under low light PFD, however, no significant differences in stomatal aperture were observed between ethanol and water-pretreated plants, with averages of 2.2 µm and 2.4 µm, respectively (Figure 4E, F, G). These results demonstrate that ethanol pretreatment can induce stomatal closure under high and medium light PFDs but not under low light PFD.

**Figure 5:**
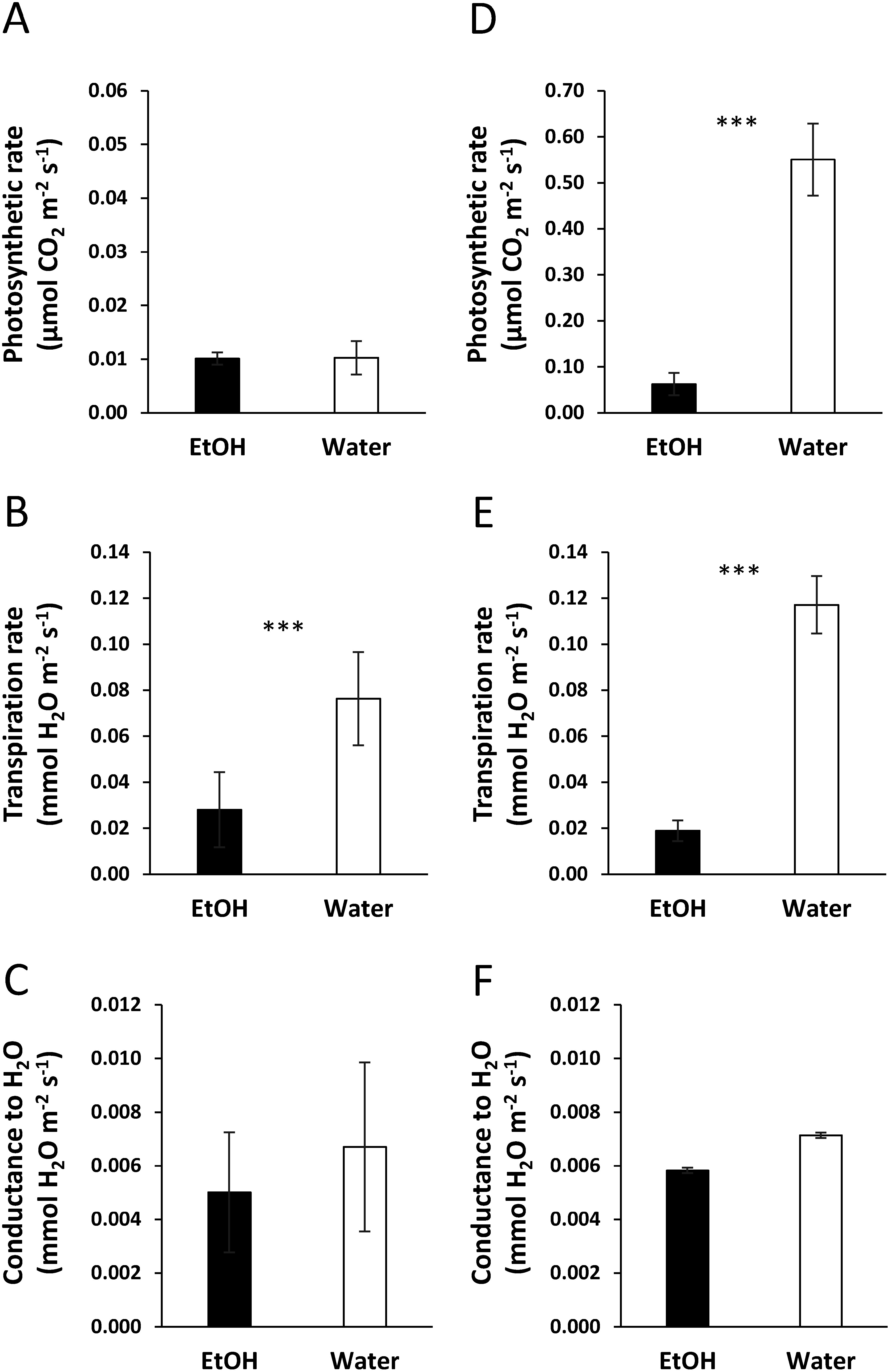
Figure 5. Effects of ethanol pretreatment on photosynthetic, transpiration rates and water conductance of third leaves below stem apices for 5 days. (A–C) Photosynthetic rate (A), transpiration rate (B), and water conductance (C) under the low light PFD conditions. (D–F) Photosynthetic rate (D), transpiration rate (E), and water conductance (F) under the high light PFD condition. *n*=6. Error bars represent the mean±standard deviation (SD). Significant differences (***, *p*≤0.01) were calculated using Student’s *t*-test.

We next assessed net photosynthetic rate, transpiration rate and water conductance of plants treated with ethanol and water under low and high light PFDs (Figure 5). Under the high light PFD, the net photosynthetic rate, transpiration rate, and water conductance rate of ethanol-pretreated plants decreased to 89%, 80%, and 19%, respectively, of those of water-pretreated plants. Under the low light PFD, these values decreased by 2%, 67%, and 15%, respectively. According to these results, ethanol pretreatment not only induces stomatal closure, but also decreases net photosynthesis under high light PFD.

### Ethanol pretreatment enhances the starch accumulation across light PFDs

**Figure 6:**
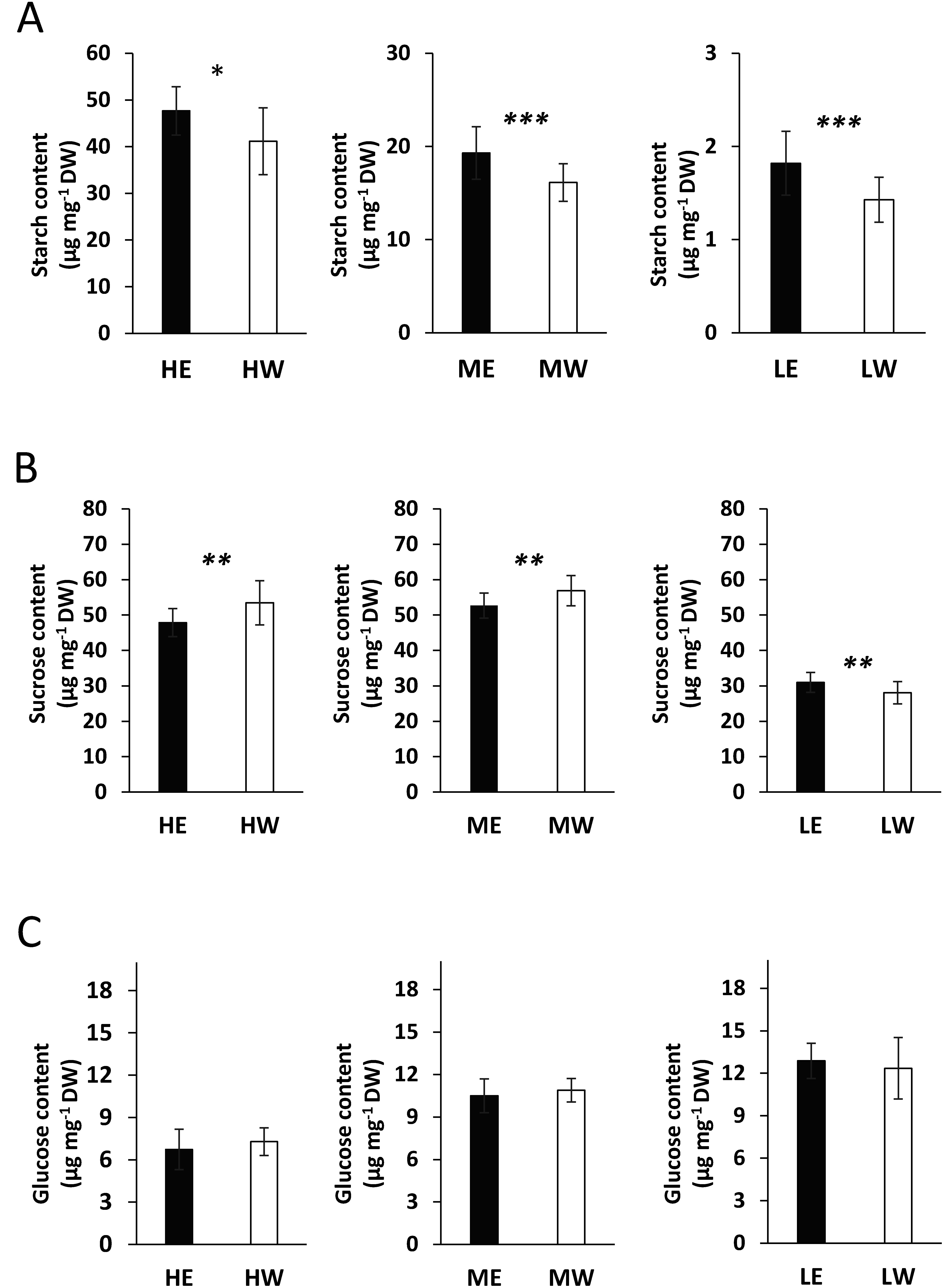
Figure 6. Starch, sucrose, and glucose contents of leaves from plants pretreated with either ethanol or water for 5 days under different light PFDs. A) Starch content. B) Sucrose content. C) Glucose content. Treatments are indicated as follows: HW, water under high light; HE, ethanol under high light; MW, water under medium light; ME, ethanol under medium light; LW, water under low light; and LE, ethanol under low light. *n*=5. Error bars represent the mean±standard deviation (SD). Significant differences (*, *p*≤0.1; **, *p*≤0.05; ***, *p*≤0.01) were calculated using Student’s *t*-test. DW represents dry weight.

Ethanol pretreatment induces gluconeogenesis in Arabidopsis leaves and promotes starch accumulation in cassava leaves, which suggests that starch serves both as a carbon source for compatible solutes and as an energy reserve during drought stress (Bashir et al. 2022; Vu et al. 2022). These findings imply that ethanol-pretreated plants may exhibit enhanced drought tolerance, potentially due to the utilization of starch reserves in the absence of soil water. To investigate whether ethanol pretreatment affects starch and sugar metabolism under varying light PFDs, we analyzed starch, sucrose, and glucose contents of leaf samples 5 days after treatment with either water or ethanol. Under high light PFD, the starch content of ethanol-pretreated plants was 47.7 µg mg^−1^ DW vs. 41.2 µg mg^−1^ DW in water-pretreated plants (Figure 6A); under medium light PFD, these values were 19.3 µg mg^−1^ DW and 16.1 µg mg^−1^ DW, respectively, and 1.8 µg mg^−1^ DW and 1.4 µg mg^−1^ DW, respectively, under low light PFD. Although starch accumulation in leaves varied with light PFD, ethanol-pretreated plants consistently exhibited higher starch accumulation than their water-pretreated counterparts. In addition, ethanol pretreatment resulted in a slight decrease in sucrose levels under high and medium light PFDs (Figure 6B), but no significant changes in glucose levels (Figure 6C) across all light PFDs, compared with water pretreatment. Our data demonstrate that ethanol pretreatment induces starch accumulation in leaves under all tested light PFDs relative to water pretreatment. In our previous study (Vu et al. 2022), we observed that ethanol pretreatment showed an accumulation of starch and slight decrease of sucrose in leaves and that after the exposure to drought stress, the ethanol-pretreated plants showed increased accumulation of sucrose and glucose than water-pretreated plants. Sucrose plays a vital role as source of energy for growth, a metabolic resource and a signaling molecule (Ruan et al. 2010). The assimilated starch may be converted into sucrose during drought stress and contribute to drought stress tolerance (Vu et al. 2022). Our results demonstrate that ethanol pretreatment under high and medium PFDs induces changes in sugar allocation, metabolism, and utilization, suggesting its contribution to the drought resistance of cassava.

### High or middle light PFDs with ethanol upregulate genes related to ABA response and HSPs

**Figure 7:**
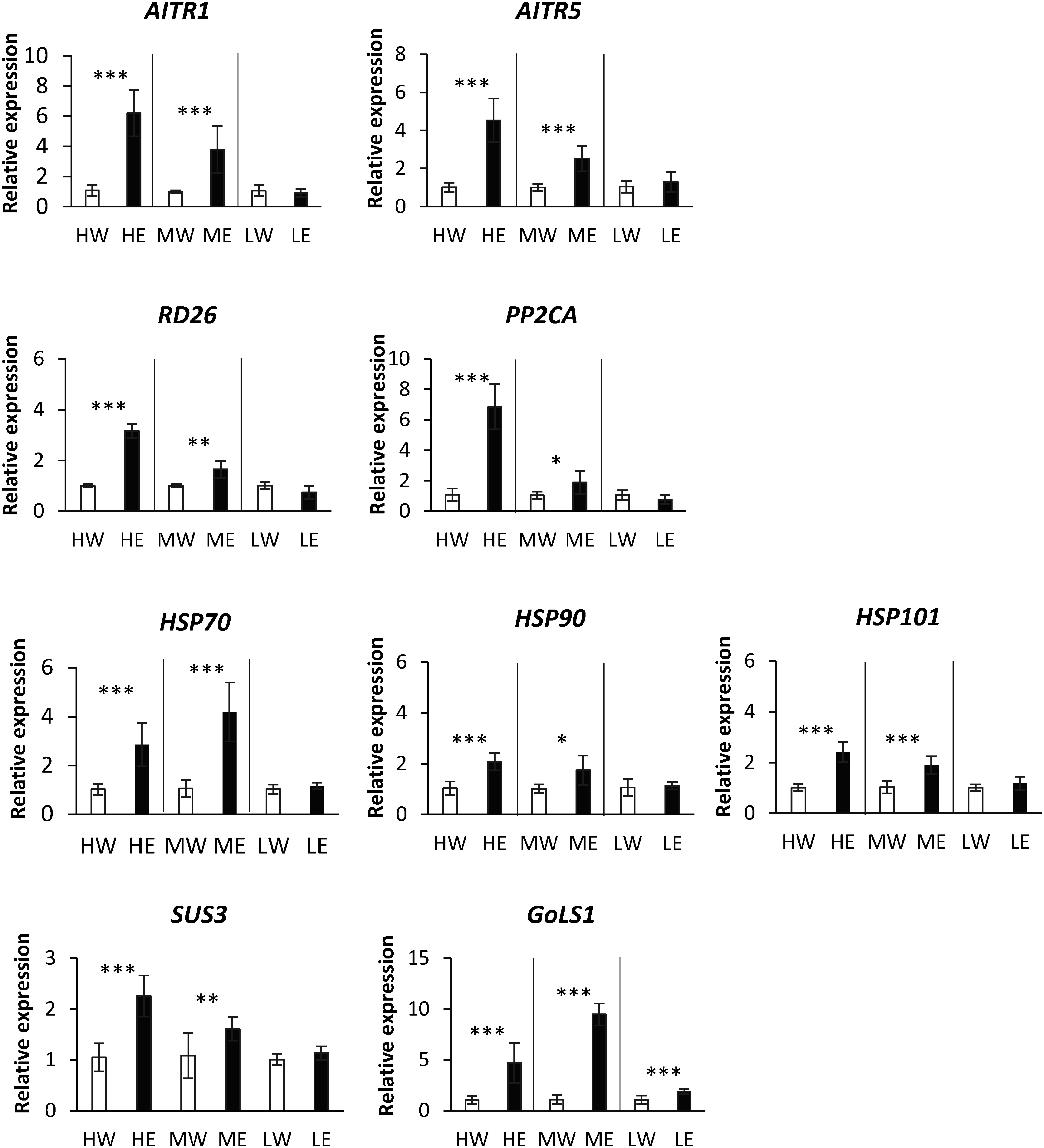
Figure 7. RT-qPCR analysis of gene expression in leaves from plants pretreated with either ethanol or water for 5 days under different light PFDs. Significant differences (*, *p*≤0.1; **, *p*≤0.05; ***, *p*≤0.01) were calculated using Student’s *t*-test, *n*=5. Error bars represent the mean±standard deviation (SD). Treatments are indicated as follows: HW, water under high light; HE, ethanol under high light; MW, water under medium light; ME, ethanol under medium light; LW, water under low light; and LE, ethanol under low light.

Previously, we demonstrated that ethanol pretreatment upregulates the expression of genes related to ABA signaling and HSPs (Vu et al. 2022). To ascertain if ethanol pretreatment enhances the expression of these genes under various light PFDs, we conducted qRT-PCR analyses on leaves from ethanol- and water-pretreated plants at five days post-treatment under high, middle, and low light PFDs. The expression of *ABA-induced transcription repressor 1* (*AITR1*) (Manes.17G106700), *AITR5* (Manes.14G054200) that functions as a feedback regulator in ABA signaling, *protein phosphatase 2C* (*PP2CA*) (Manes.07G119400) that functions as one of the key central regulators involved in ABA signaling, *NAC domain transcriptional regulator superfamily protein* (*RD26*) (Manes.15G084800) that functions as a transcriptional activator in ABA-mediated dehydration response, *sucrose synthase 3* (*SUS3*) that catalyze both the synthesis of sucrose from fructose and UDP-glucose and cleavage of sucrose, and the genes encoding heat shock protein (HSPs), such as heat shock protein HSP70 (Manes.11G067500), HSP90 (Manes.14G022300) and HSP101 (Manes.06G085600) was higher in ethanol-pretreated plants than in water-pretreated plants under high and middle light PFDs (Figure 7). However, under low light PFDs, no significant differences were observed in the expression levels of these genes (Figure 7). The expression of an osmoprotectant-related gene, *galactinol synthase 1* (*GolS1*) (Manes.05G012000) was higher in ethanol-pretreated plants across all light PFDs (Figure 7).

In middle and high light PFDs, the ABA signaling pathway, HSP chaperone network, and starch accumulation processes were activated. In contrast, under low light PFD, only starch metabolism was active (Figures 6 and 7). These findings indicate that middle and high light PFDs, combined with ethanol pretreatment, are crucial for activating these pathways and enhancing drought tolerance. Conversely, low light PFD combined with ethanol pretreatment does not significantly enhance drought tolerance, as it fails to activate the pathways associated with ABA signaling and the HSP chaperone network (Figure 7). Exogenous ethanol application significantly enhances drought tolerance by regulating ABA signaling and promoting stomatal closure to conserve water (Bashir et al. 2022). Light-induced heat acts as a trigger for the induction of HSP gene expression, which are referred to as heat-induced molecular chaperones (Clément et al. 2011). These chaperones, especially HSP70 and HSP90, play a pivotal role in the mechanisms that initiate acquired thermotolerance in land plants (Serrano et al. 2019; Song et al. 2012).

### Assessment of ethanol pretreatment in relation to senescence and drought stress responses

To examine whether ethanol pretreatment is associated with drought stress or aging (the term ‘senescence’ may be more appropriate), we analyzed the expression levels of *senescence-associated gene 12* (*SAG12*, At5g45890) (Gan and Amasino 1995) and transcription factor *WRKY53* (At4g23810) (Miao et al. 2008), that are well-known senescence markers and were identified by previous cassava transcriptome analysis (Vu et al. 2022) that has been performed under high light PFDs. The results showed that cassava *SAG12* and *WRKY53* genes were not upregulated by ethanol pretreatment and the following drought treatment (Supplementary Table S2). However, the transcriptome analysis revealed that ethanol pretreatment upregulates the expression of *Bcl-2-associated athanogene 5* (*BAG5*) (Supplementary Table S2), whose increased expression promotes plant senescence (Li et al. 2016). Our previous analysis showed ethanol treatment increased accumulation of assimilated starch in leaves and the following drought treatment increased accumulation of glucose and sucrose (Vu et al. 2022) that induce leaf senescence (Wingler et al. 2006). Thus, senescence might progress mildly during ethanol pretreatment and the following drought treatment.

## Conclusions

In this study, we discovered that ethanol pretreatment at the light PFDs exceeding 60 µmol photons m^−2^ s^−1^ is more effective for ethanol-mediated drought avoidance, through the upregulation of three pathways: ABA signaling, metabolism of starch and sugar, and HSP chaperones. Under high PFD, excess light energy may lead to stress, thereby reducing ethanol’s effectiveness. Conversely, low PFD (∼4 µmol photons m^−2^ s^−1^) did not enhance ethanol-mediated drought avoidance in cassava. These results suggest that molecular mechanisms related to light perception are involved in the switch that enhances ethanol-mediated drought avoidance. We propose that photobody formation, controlled by liquid–liquid phase separation, might be involved in the switching (Chen et al. 2022). Ethanol application, particularly in conjunction with natural environmental light and heat, is expected to exert physiological effects on plants. These insights contribute to a better understanding of the biological implications of ethanol treatment, particularly its impact on maintaining water content in cassava plants when combined with light and heat.

## Abbreviations

ABA: abscisic acid
HSP: heat shock protein
MES: 2-morpholinoethanesulfonic acid monohydrate
PCR: polymerase chain reaction
PFD: photon flux density
RWC: relative water content

## Description of supplementary files

Supplementary Figure S1. Light wavelength spectra under different light PFDs.

Supplementary Figure S2. Representative phenotypes of cassava plants after pretreatment with 1% ethanol or water for 5 days followed by drying for 6 days under different light PFDs.

Supplementary Table S1. Primer sequences for qRT-PCR.

Supplementary Table S2. Expression data of cassava transcriptome analysis performed during ethanol pretreatment and the following drought stress treatments.

## References

[RBashir2022] Bashir K, Todaka D, Rasheed S, Matsui A, Ahmad Z, Sako K, Utsumi Y, Vu AT, Tanaka M, Takahashi S, et al. (2022) Ethanol-mediated novel survival strategy against drought stress in plants. *Plant Cell Physiol* 63: 1181–1192 36003026 10.1093/pcp/pcac114 PMC9474946

[RBashir2025] Bashir K, Todaka D, Sako K, Ueda M, Aziz F, Seki M (2025) Chemical application improves stress resilience in plants. *Plant Mol Biol* 115: 47 40105987 10.1007/s11103-025-01566-w PMC11922999

[RCasal2013] Casal JJ (2013) Photoreceptor signaling networks in plant responses to shade. *Annu Rev Plant Biol* 64: 403–427 23373700 10.1146/annurev-arplant-050312-120221

[RChen2022] Chen D, Lyu M, Kou X, Li J, Yang Z, Gao L, Li Y, Fan LM, Shi H, Zhong S (2022) Integration of light and temperature sensing by liquid-liquid phase separation of phytochrome B. *Mol Cell* 82: 3015–3029.e6 35728588 10.1016/j.molcel.2022.05.026

[d67e821] Clément M, Leonhardt N, Droillard MJ, Reiter I, Montillet JL, Genty B, Laurière C, Nussaume L, Noël LD (2011) The cytosolic/nuclear HSC70 and HSP90 molecular chaperones are important for stomatal closure and modulate abscisic acid-dependent physiological responses in Arabidopsis. *Plant Physiol* 156: 1481–1492 21586649 10.1104/pp.111.174425 PMC3135925

[REl-Sharkawy2004] El-Sharkawy MA (2004) Cassava biology and physiology. *Plant Mol Biol* 56: 481–501 15669146 10.1007/s11103-005-2270-7

[RFahad2017] Fahad S, Bajwa AA, Nazir U, Anjum SA, Farooq A, Zohaib A, Sadia S, Nasim W, Adkins S, Saud S, et al. (2017) Crop production under drought and heat stress: Plant responses and management options. *Front Plant Sci* 8: 1147 28706531 10.3389/fpls.2017.01147 PMC5489704

[RGan1995] Gan S, Amasino RM (1995) Inhibition of leaf senescence by autoregulated production of cytokinin. *Science* 270: 1986–1988 8592746 10.1126/science.270.5244.1986

[RHu2016] Hu M, Hu W, Xia Z, Zhou X, Wang W (2016) Validation of reference genes for relative quantitative gene expression studies in cassava (*Manihot esculenta* Crantz) by using quantitative Real-Time PCR. *Front Plant Sci* 7: 680 27242878 10.3389/fpls.2016.00680 PMC4871855

[RLi2016] Li L, Xing Y, Chang D, Fang S, Cui B, Li Q, Wang X, Guo S, Yang X, Men S, et al. (2016) CaM/BAG5/Hsc70 signaling complex dynamically regulates leaf senescence. *Sci Rep* 6: 31889 27539741 10.1038/srep31889 PMC4990970

[RMalik2020] Malik AI, Kongsil P, Nguyễn VA, Ou W, Sholihin, Srean P, Sheela MN, Becerra López-Lavalle LA, Utsumi Y, Lu C, et al. (2020) Cassava breeding and agronomy in Asia: 50 years of history and future directions. *Breed Sci* 70: 145–166 32523397 10.1270/jsbbs.18180 PMC7272245

[RMatthews2020] Matthews JSA, Vialet-Chabrand S, Lawson T (2020) Role of blue and red light in stomatal dynamic behaviour. *J Exp Bot* 71: 2253–2269 31872212 10.1093/jxb/erz563 PMC7134916

[RMiao2008] Miao Y, Smykowski A, Zentgraf U (2008) A novel upstream regulator of WRKY53 transcription during leaf senescence in *Arabidopsis thaliana.* *Plant Biol (Stuttg)* 10(Suppl 1): 110–120 18721316 10.1111/j.1438-8677.2008.00083.x

[RMuiruri2021] Muiruri SK, Ntui VO, Tripathi L, Tripathi JN (2021) Mechanisms and approaches towards enhanced drought tolerance in cassava (*Manihot esculenta*). *Curr Plant Biol* 28: 100227

[RNakashima2014] Nakashima K, Yamaguchi-Shinozaki K, Shinozaki K (2014) The transcriptional regulatory network in the drought response and its crosstalk in abiotic stress responses including drought, cold, and heat. *Front Plant Sci* 5: 170 24904597 10.3389/fpls.2014.00170 PMC4032904

[RNishiyama2011] Nishiyama R, Watanabe Y, Fujita Y, Le DT, Kojima M, Werner T, Vankova R, Yamaguchi-Shinozaki K, Shinozaki K, Tran LSP, et al. (2011) Analysis of cytokinin mutants and regulation of cytokinin metabolic genes reveals important regulatory roles of cytokinins in drought, salt and abscisic acid responses, and abscisic acid biosynthesis. *Plant Cell* 23: 2169–2183 21719693 10.1105/tpc.111.087395 PMC3160038

[RRahman2022] Rahman MM, Mostofa MG, Das AK, Anik TR, Keya SS, Ahsan SM, Khan MAR, Ahmed M, Rahman MA, Tran LSP, et al. (2022) Ethanol positively modulates photosynthetic traits, antioxidant defense and osmoprotectant levels to enhance drought acclimatization in soybean. *Antioxidants* 11: 516 35326166 10.3390/antiox11030516 PMC8944470

[RRoeber2021] Roeber VM, Bajaj I, Rohde M, Schmülling T, Cortleven A (2021) Light acts as a stressor and influences abiotic and biotic stress responses in plants. *Plant Cell Environ* 44: 645–664 33190307 10.1111/pce.13948

[RRuan2010] Ruan YL, Jin Y, Yang YJ, Li GJ, Boyer JS (2010) Sugar input, metabolism, and signaling mediated by invertase: Roles in development, yield potential, and response to drought and heat. *Mol Plant* 3: 942–955 20729475 10.1093/mp/ssq044

[RSako2021] Sako K, Nagashima R, Tamoi M, Seki M (2021) Exogenous ethanol treatment alleviates oxidative damage of *Arabidopsis thaliana* under conditions of high-light stress. *Plant Biotechnol (Tokyo)* 38: 339–344 34782821 10.5511/plantbiotechnology.21.0715a PMC8562572

[RSako2020] Sako K, Nguyen HM, Seki M (2020) Advances in chemical priming to enhance abiotic stress tolerance in plants. *Plant Cell Physiol* 61: 1995–2003 10.1093/pcp/pcaa119 32966567

[RSerrano2019] Serrano N, Ling Y, Bahieldin A, Mahfouz MM (2019) Thermopriming reprograms metabolic homeostasis to confer heat tolerance. *Sci Rep* 9: 181 30655560 10.1038/s41598-018-36484-z PMC6336788

[RSong2012] Song L, Jiang Y, Zhao H, Hou M (2012) Acquired thermotolerance in plants. *Plant Cell Tissue Organ Cult* 111: 265–276

[RTallman2004] Tallman G (2004) Are diurnal patterns of stomatal movement the result of alternating metabolism of endogenous guard cell ABA and accumulation of ABA delivered to the apoplast around guard cells by transpiration? *J Exp Bot* 55: 1963–1976 15310824 10.1093/jxb/erh212

[RUtsumi2012] Utsumi Y, Tanaka M, Morosawa T, Kurotani A, Yoshida T, Mochida K, Matsui A, Umemura Y, Ishitani M, Shinozaki K, et al. (2012) Transcriptome analysis using a high-density oligomicroarray under drought stress in various genotypes of cassava: An important tropical crop. *DNA Res* 19: 335–345 22619309 10.1093/dnares/dss016 PMC3415295

[RUtsumi2011] Utsumi Y, Utsumi C, Sawada T, Fujita N, Nakamura Y (2011) Functional diversity of isoamylase oligomers: The ISA1 homo-oligomer is essential for amylopectin biosynthesis in rice endosperm. *Plant Physiol* 156: 61–77 21436381 10.1104/pp.111.173435 PMC3091037

[RUtsumi2019] Utsumi Y, Utsumi C, Tanaka M, Ha CV, Takahashi S, Matsui A, Matsunaga TM, Matsunaga S, Kanno Y, Seo M, et al. (2019) Acetic acid treatment enhances drought avoidance in cassava (*Manihot esculenta* Crantz). *Front Plant Sci* 10: 521 31105723 10.3389/fpls.2019.00521 PMC6492040

[RVu2022] Vu AT, Utsumi Y, Utsumi C, Tanaka M, Takahashi S, Todaka D, Kanno Y, Seo M, Ando E, Sako K, et al. (2022) Ethanol treatment enhances drought stress avoidance in cassava (*Manihot esculenta* Crantz). *Plant Mol Biol* 110: 269–285 35969295 10.1007/s11103-022-01300-w

[RWingler2006] Wingler A, Purdy S, MacLean JA, Pourtau N (2006) The role of sugars in integrating environmental signals during the regulation of leaf senescence. *J Exp Bot* 57: 391–399 16157653 10.1093/jxb/eri279

