# Supplementary material for: Light photon flux density affects ethanol-mediated drought avoidance in cassava (Manihot esculenta Crantz)
Source: Plant Biotechnol (Tokyo). 2025 Dec 25;42(4):413–22. doi: 10.5511/plantbiotechnology.25.0426a (PMC12781900; doi:10.5511/plantbiotechnology.25.0426a)
Supplement: Supplementary Data [file plantbiotechnology-42-4-25.0426a-s001.pdf]

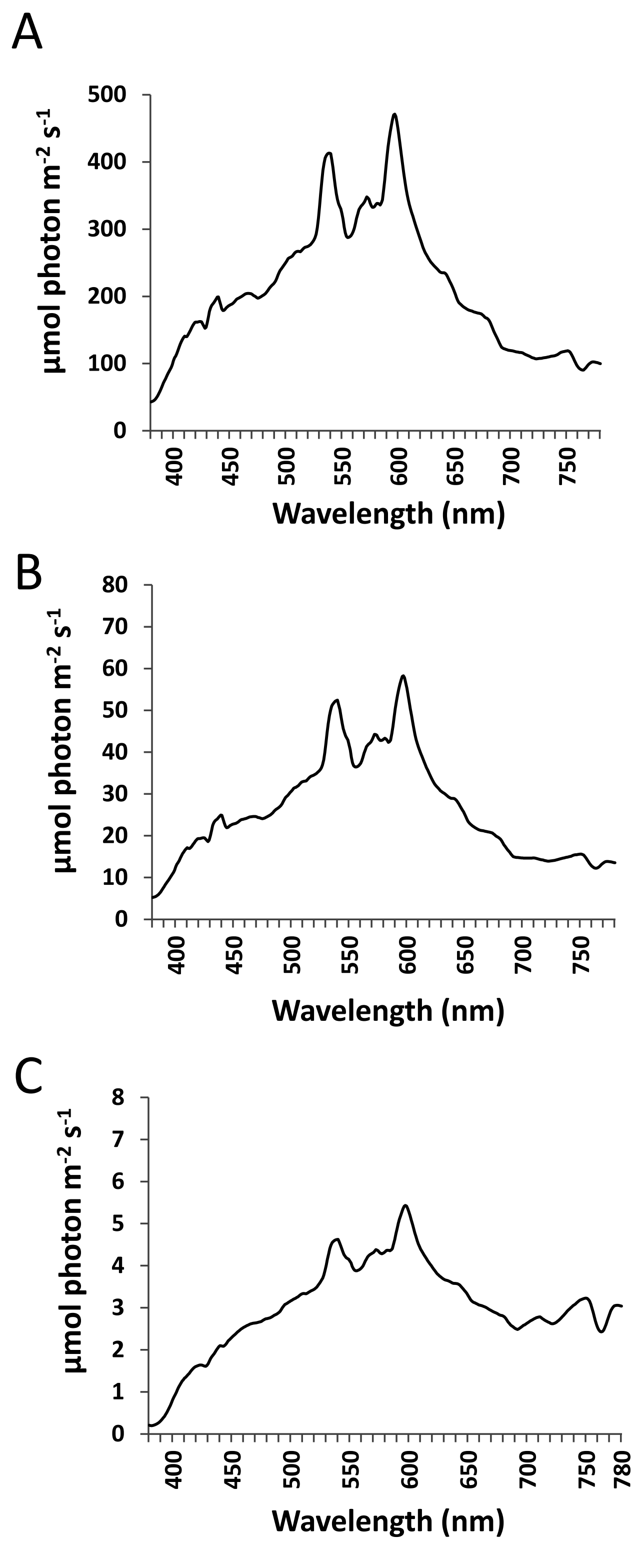

Supplementary Figure S1. Light wavelength spectra under different light PFDs.

A) High light PFD. B) Medium light PFD. C) Low light PFD.

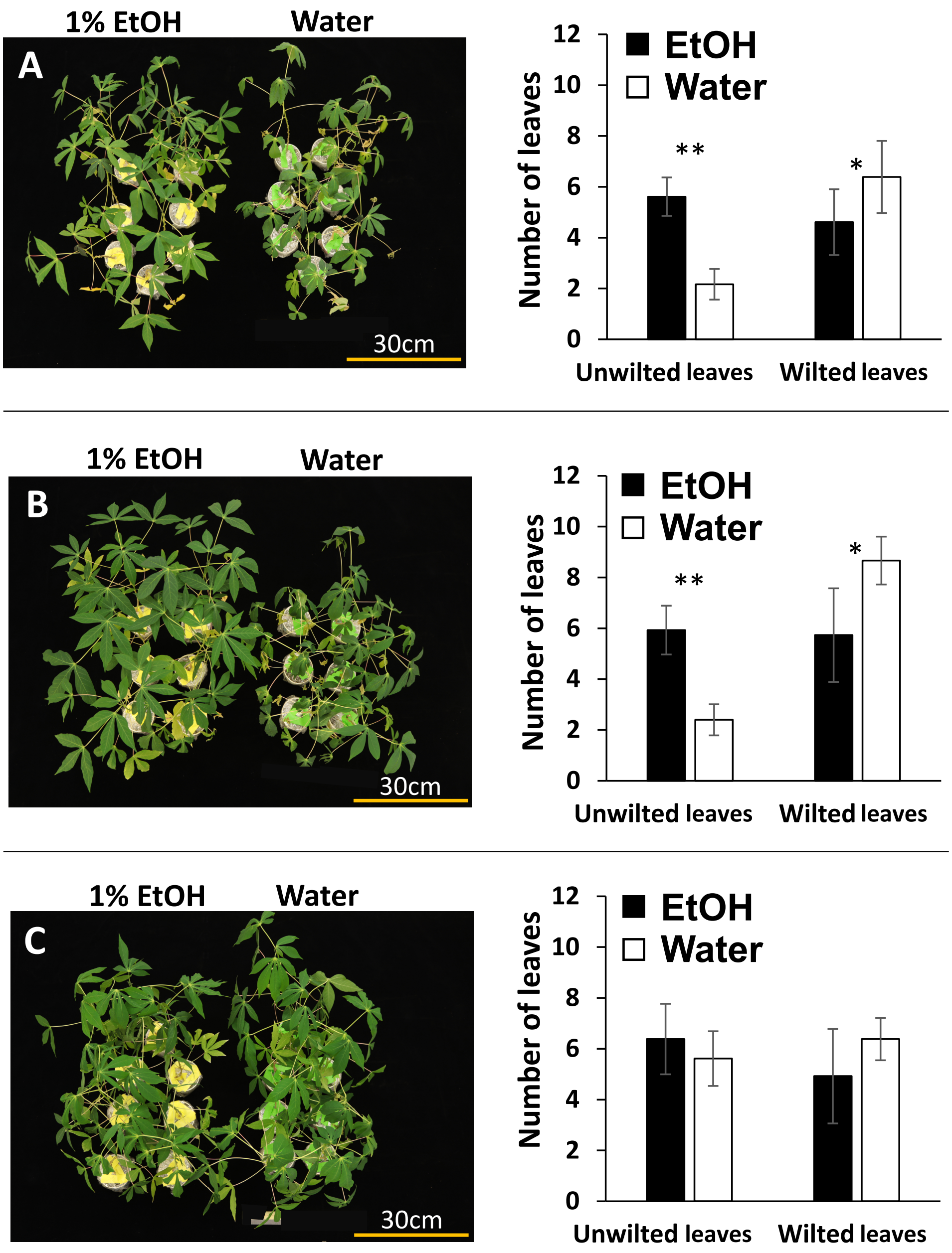

Supplementary Figure S2. Representative phenotypes of cassava plants after pretreatment with 1% ethanol or water for 5 days followed by drying for 6 days under different light PFDs. Scale bar = 30 cm, n = 12. Error bars represent the mean ± standard deviation (SD). Significant differences (**, $P \leq 0.01$ and *, $P \leq 0.05$) were calculated using Student's *t*-test. Panels A, B, and C represent plants under high, medium, and low light PFDs, respectively.

Supplementary Table S1. Primer sequences for qRT-PCR

Primer name	Cassava gene model	Sequences (5'-3')		Size (bp)
		Forward	Reverse	
AITR1	Manes.17G106700	CATTCTCTCAATAGGGCGAGTC	GGGTGCAATTATAGGCGTAGTG	87
AITR5	Manes.14G054200	AGATGATTGCCAGGTGGAAG	GCTAAAACATGGAAGGACAGC	133
AREB1	Manes.18G037900	ATCCCTTGCCTGTTCTTCG	GGAGACATTAGCCAAACATGC	89
RD26	Manes.15G084800	TGTACCATTCTTTGGGGATATG	AAAAGGCCCCTATCAACTCC	77
PP2CA	Manes.07G119400	TCTTGGGTATTGGACTCCTCTC	GCCTGCATTTCCTGATGTG	134
HAI3	Manes.02G128000	CGATGTTGCTGACGAAGTTG	CCTGAAATCAAGACCTCAGTCC	84
HSP70	Manes.11G067500	ACTTGAGGCCTTGTGCAATC	TAGTCTCCGTTCGGCATTTC	99
HSP90	Manes.14G022300	TTCTTGTCAGCTGCATGGTC	CTGGGAGTCGTTCAAATGAG	149
HSP101	Manes.06G085600	AGTGTCCCAAGGAATGATGC	TTGTACGATCCCCGATTGTC	109
SUS3	Manes.01G221900	AAGGAGAATCCAAGCTGCTG	ACCCCAGCCAATGTCAATAG	110
GoLS1	Manes.05G012000	TTAGTGGTGGCGGTTTTACC	TTCAATCTCCCGGACTAAGC	84

Supplementary Table S2. Expression data of cassava transcriptome analysis performed during ethanol pretreatment and the following drought stress treatments[a].

Probe	E5/W5 [a]			E5D6/W5D6 [a]			E5D12/W5D12 [a]			Description [b]	Gene name	AGI code [c]	Cassava gene model [d]
	log_2FC	P-value by student t-test	adjusted P-value by BH	log_2FC	P-value by student t-test	adjusted P-value by BH	log_2FC	P-value by student t-test	adjusted P-value by BH				
RknMes02_036284	0.0	0.726	0.952	0.1	0.324	0.677	0.0	0.954	0.987	BCL-2-associated athanogene 5	ATBAG5,BAG5	AT1G12060	Manes.10G111700
RknMes02_039767	3.1	0.000	0.000	0.7	0.069	0.336	-0.3	0.332	0.655	BCL-2-associated athanogene 5	ATBAG5,BAG5	AT1G12060	Manes.13G007700
RknMes02_002628	-0.6	0.072	0.526	-0.7	0.033	0.228	0.2	0.497	0.778	WRKY family transcription factor	ATWRKY53,WRKY53	AT4G23810	Manes.01G047200
RknMes02_014838	0.0	0.796	0.966	-0.2	0.152	0.487	0.3	0.053	0.267	WRKY family transcription factor	ATWRKY53,WRKY53	AT4G23810	Manes.05G030900
RknMes02_038123	-0.5	0.345	0.813	0.8	0.113	0.425	1.4	0.006	0.080	WRKY family transcription factor	ATWRKY53,WRKY53	AT4G23810	Manes.01G253400
RknMes02_009793	-0.1	0.737	0.954	0.7	0.026	0.202	-0.2	0.471	0.762	senescence-associated gene 12	SAG12	AT5G45890	Manes.15G123400
RknMes02_019794	0.0	0.876	0.981	-0.3	0.030	0.218	0.0	0.977	0.993	senescence-associated gene 12	SAG12	AT5G45890	Manes.16G038400
RknMes02_039819	0.0	0.779	0.963	0.1	0.401	0.738	-0.1	0.391	0.705	senescence-associated gene 12	SAG12	AT5G45890	Manes.16G037600
RknMes02_051098	0.0	0.869	0.980	0.0	0.959	0.990	0.4	0.057	0.280	senescence-associated gene 12	SAG12	AT5G45890	Manes.17G054300
RknMes02_053914	-0.1	0.704	0.948	-0.1	0.660	0.883	-0.3	0.083	0.339	senescence-associated gene 12	SAG12	AT5G45890	Manes.16G037700
RknMes02_054166	-0.1	0.482	0.878	0.1	0.120	0.436	-0.1	0.164	0.470	senescence-associated gene 12	SAG12	AT5G45890	Manes.04G163000
RknMes02_054254	0.2	0.134	0.631	0.3	0.030	0.219	0.4	0.013	0.125	senescence-associated gene 12	SAG12	AT5G45890	Manes.13G139300

[a] Cassava transcriptome analysis using a custom oligomicroarray (Vu et al. 2022) was performed using the leaf samples from the plants pretreated with 1.0% ethanol for 5 days (E5), the samples pretreated with water for 5 days (W5), the samples from the plants exposed to drought stress for 6 days after the pretreatment with 1.0% ethanol for 5 days (E5D6), the samples from the plants exposed to drought stress for 6 days after the pretreatment with water for 5 days (W5D6), the samples from the plants exposed to drought stress for 12 days after the pretreatment with 1.0% ethanol for 5 days (E5D12), the samples from the plants exposed to drought stress for 12 days after the pretreatment with water for 5 days (W5D12).

[b] Encoded proteins/other features indicate the putative functions of the gene products that are expected from sequence similarity. The information for the NCBI protein reference sequence with the highest sequence similarity with the probes is shown.

[c] The information for AGI locus ID with the highest sequence similarity with the probe is shown.

[d] The information for cassava gene code with the highest sequence similarity with the probe is shown.

Reference

Vu AT, Utsumi Y, Utsumi C, Tanaka M, Takahashi S, Todaka D, Kanno Y, Seo M, Ando E, Sako K et al. (2022) Ethanol treatment enhances drought stress avoidance in cassava (Manihot esculenta Crantz). Plant Mol Biol 110(3): 269–285
